# Seroprevalence of *Fasciola* *gigantica* infection in bovines using cysteine proteinase dot enzyme-linked immunosorbent assay

**DOI:** 10.14202/vetworld.2017.1189-1193

**Published:** 2017-10-06

**Authors:** Niranjan Kumar, Anju Varghese, J. B. Solanki

**Affiliations:** Department of Parasitology, College of Veterinary Science and Animal Husbandry, Navsari Agricultural University, Navsari - 396 450, Gujarat, India

**Keywords:** bovines, cysteine proteinase, dot enzyme-linked immunosorbent assay, *Fasciolagigantica*

## Abstract

**Aim:**

The objective of the present study was to know the seroprevalence status of *Fasciola gigantica* infection in cattle and buffaloes using cysteine proteinase (CP) antigen in dot enzyme-linked immunosorbent assay (ELISA) format under field conditions.

**Materials and Methods:**

As per the standard protocol, the sera were collected from the blood of 112 cattle and 38 buffaloes of coastal areas of Navsari district, South Gujarat, India. The indirect ELISA was performed on the strip of nitrocellulose paper blotted with 1 µl of CP antigen, to detect *F*. *gigantica* seropositive animals.

**Results:**

The native CP of *F*. *gigantica* revealed a single visible band on 10% sodium dodecyl sulfate-polyacrylamide gel electrophoresis. There was no any noted cross-reaction between the selected antigen and sera of *Gastrothylax crumenifer*-infected animals in ELISA. Out of 150 screened bovines, the sera of 47 (31.33%) were found to be reactive in dot-ELISA, with a prevalence rate of 31.25% and 31.58% in cattle and buffaloes, respectively. The seropositive bovines with heavy, moderate, and light level of infection were 44.68%, 34.04%, and 21.28%, respectively (p<0.05 between heavy and light; p>0.05 between moderate and heavy or light). The share of *F*. *gigantica* seropositive and negative animals was 31% and 69%, respectively. The optical density at 450 nm of pooled sera of seropositive bovines with heavy, moderate, and light reactivity in plate-ELISA was significantly higher with field or reference ­negative sera.

**Conclusion:**

The CP-based dot-ELISA can be useful for field veterinarians for quick and timely isolation of the animals requiring urgent flukicide therapy.

## Introduction

Fasciolosis, the snail-borne disease, is widely prevalent in most geographical regions of the world. The infection rate caused by tropical fluke, *Fasciola gigantica*, varies between 10 to 80% among buffalo and cattle (as primary hosts), followed by sheep and goat [1-3]. The disease is recognized as one of the most economically important helminth infection of the ruminants [4], with worldwide monetary losses conservatively estimated at over US $3.2 billion per annum [5]. The economic losses are due to mortality, morbidity, poor growth, and productivity loss in the infected animals [6], and hence, the prevention and control of fasciolosis could contribute significantly to improve animal production [7].

The feasible approaches to prevent and control the fasciolosis are based on reducing the population of the snail intermediate host or its timely identification followed by drug dosing in the definitive host [8]. Diagnosis of animal fasciolosis largely based on the microscopic demonstration of the parasite egg in the feces on 12-14 weeks postinfection (WPI), i.e., only possible in patent/late phase of infection [9]. Furthermore, the low sensitivity of the coprological methods resulted in the underdiagnosis of weak infection, and thus, it is not suitable for analyzing the disease condition in the large herds [10]. The pathogen causes major harm to the definitive host during its prepatent/early phase of infection [11]. Therefore, diagnosis in the prepatent/early phase of infection is the major objective to ensure sustainability in animal production.

Serological assays are the only options for the detection of infection at an early prepatent stage using parasite-specific antigenic moieties [12]. However, the helminths are a complex organism and are known to share antigenic epitopes, resulting in cross-reaction and false-positive case [13]. Therefore, to develop a serological assay for the diagnosis of fasciolosis in the ruminants, continuous efforts are needed to identify and characterize the antigen for their utilization under field conditions for the early and specific detection of the infection [14-18]. Furthermore, specific diagnosis will help in the selection of a specific drug for the treatment and control of the disease, and thus, minimizes drug abuse, often leading into the development of resistant strains of the parasites [19].

An attempt to develop *F*. *gigantica*-specific diagnostic kit for the bovine, the Helminthology Laboratory, Division of Parasitology, Indian Veterinary Research Institute, Izatnagar - 243 122 had isolated and characterized the 28 kDa cysteine proteinase (CP) from the excretory-secretory product of the fluke [16]. This antigen-based enzyme-linked immunosorbent assay (ELISA) successfully detected fasciolosis in the buffaloes with a high level of sensitivity and specificity under field situations [12,17,20]. The present study aimed to know the seroprevalence status of *F*. *gigantica* infection in the bovines using CP antigen in dot-ELISA format under field conditions of heavy rained areas of South Gujarat of Navsari district, India.

## Materials and Methods

### Ethical approval

As the study was conducted with the clinical cases, so ethical committee approval was not required.

### Study area

The villages of Navsari (20.95°N, 72.93°E) district of Gujarat state of India are characterized with average monthly rainfall of 0 to 1663.77±448.00 mm in winter/summer to rainy season and the relative humidity from 29.2±4.25 to 84.73±3.44% in February to August.

### Biological sample collection and processing

Each 5 ml of blood was collected from the jugular vein of properly restrained 112 cattle and 38 buffaloes, presented before the clinician for disease diagnosis/treatment in the clinical camps organized in the above mentioned areas, using sterile needle and syringe. The collected blood samples were allowed to coagulate and further centrifuged to obtain the sera and stored at −20°C until further analysis.

### Reference sera and CP antigen for ELISA

Five positive/negative reference sera and diagnostic antigen, CP of *F. gigantica* infection were provided by the Helminthology Laboratory, Division of Parasitology, IVRI, Izatnagar. The negative control sera were originated from the buffalo calves reared under experimental conditions for 1 year, properly dewormed, and proven as free from *F. gigantica* infection by the expert. Simultaneously, the *F*. *gigantica* free buffalo calves were infected with the viable metacercariae and bled at 8^th^ week postinfection to isolate positive control sera. The CP antigen was purified by the said laboratory from the *in vitro* released *F*. *gigantica* excretory-secretory products by two-step alcohol fractionation, followed by ion-exchange chromatography [16]. The integrity of the diagnostic *F*. *gigantica*-specific CP antigen was checked on 10% sodium dodecyl sulfate-polyacrylamide gel electrophoresis (SDS-PAGE) [16]. The said laboratory had also provided each five *F*. *gigantica*-positive/negative and *Gastrothylax* *crumenifer*-positive/negative buffalo’s field sera, proven by postmortem followed by a fecal examination. The reactivity pattern of these sera was used to compare the positive and negative animals in ELISA format with CP antigen.

### Seroprevalence study

The indirect dot-ELISA was performed as per the method described by Sriveny *et al*. [17] with adjustable modifications. Briefly, CP antigen of 1 µl (~200 ng) was blotted onto the rectangular strips of nitrocellulose paper (Biorad, California, USA). The antigen blotted spots were air-dried, incubated at 37°C for 1 h, and stored at room temperature until required. The strips were thoroughly washed 3 times in phosphate buffer saline (PBS)-Tween-20 (0.05%, 50 µl of Tween-20 in 100 ml of PBS of pH 7.2) wash buffer. The strips were blocked in a solution containing 5% skimmed milk powder in PBS for 2 h at room temperature. The strips were thoroughly washed 3 times with wash buffer, and then, incubated in 1:50 diluted sera in 1% skimmed milk in PBS separately for 1 h at room temperature. After three washings, the strips were dipped in rabbit anti-bovine immunoglobulin G (IgG)-horse radish peroxidase (HRPO) conjugate (Santa Cruz, USA) at 1:2000 dilution in 1% skimmed milk in PBS for 1 h at room temperature. Following five washes, the strips were incubated at room temperature in the dark in diaminobenzidine (Sigma Chemical Company, USA) chromogenic substrate buffer (8 mg diaminobenzidine, 10 ml PBS, 10 µl H_2_O_2_, for a total 10 ml substrate volume) for color development. The strips were thoroughly washed after visible dot formation in the several changes of the distilled water to stop further chromogenic reactions and dried on the filter paper. The positive sera of *F*. *gigantica* infection were characterized by the formation of distinct brown color dots on the paper while negative sera showed no dots. Depending on the intensity of the dot, the bovines were grouped into heavily, moderately, and lightly infected with *F*. *gigantica*. The sera were pooled accordingly to form heavily, moderately, lightly, and negatively *F*. *gigantica*-infected one, and further, analyzed in the indirect plate-ELISA.

Indirect plate-ELISA was performed as per the method described by Kumar *et al*. [14,15] with some modifications. The flat-bottom polystyrene microtiter plates (Greiner, Germany) were incubated overnight at 4°C with 100 µl/well of carbonate and bicarbonate coating buffer containing 5.0 µg/ml of CP antigen. The wells were washed twice for 5 min with washing buffer and incubated at 37°C for 2 h with 300 µl/well of blocking solution, 3% skimmed milk in PBS. After thrice washings of the plate in the washing buffer, each well was loaded with 100 µl of 1:100 diluted pooled sera in 1% skimmed milk prepared in PBS and incubated at 37°C for 1 h. Wells were washed again 3 times with wash buffer, and 100 µl of rabbit anti-bovine IgG-HRPO (Santa Cruz, USA) conjugate in 1:5000 dilution prepared in 1% skimmed milk in PBS was added to each well and incubated at 37°C for 1 h. After five vigorous washings, 100 µl of chromogenic substrate with O-phenylenediamine dihydrochloride (OPD) (Sigma, USA) solution (OPD - 0.8 mg/ml phosphate-citrate buffer pH 5.0 and 1 µl H_2_O_2_/ml substrate buffer) was added to each well and kept in the dark for color development. The reaction was stopped after the development of appropriate chromogenic reaction using 50 µl 3 N HCl/well. The absorbance readings were taken at 450 nm on an ELISA reader (Biorad, USA). The data expressed as the mean of the optical density (OD) were recorded for the grouped samples.

### Statistical analysis

The results were compiled systematically, and data were analyzed using IBM SPSS Statistics 20.00 for Windows (SPSS Inc., Chicago, USA) to perform one-way ANOVA using Duncan for determination of statistical significance. The p>0.05 was considered as statistically non-significant.

## Results

**Figure-1:**
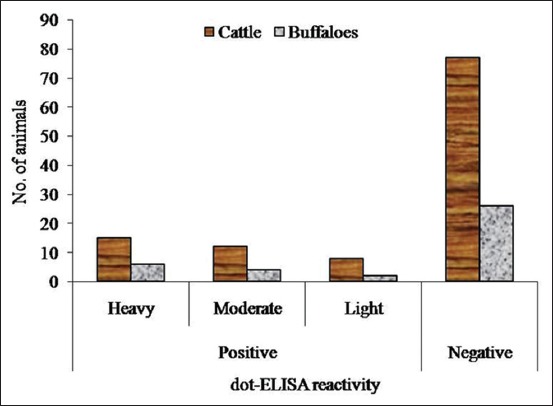
Dot enzyme-linked immunosorbent assay reactivity pattern of *Fasciola* *gigantica* infection.

**Figure-2:**
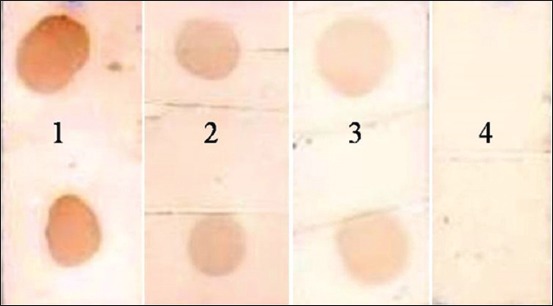
Representative dot strip no. 1-3 (1 - heavy, 2 - moderate, and 3 - light) and 4 showing positive and negative reactivity of *Fasciola* *gigantica* infection, respectively.

**Figure-3:**
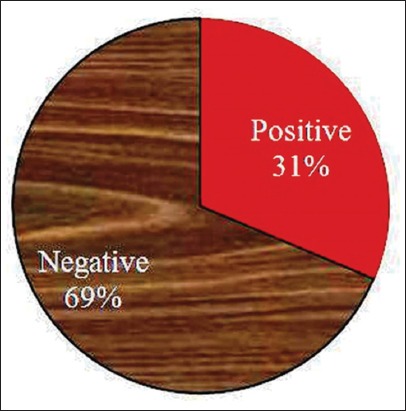
Percent distribution of *Fasciola* *gigantica* sero-positive/negative bovines.

**Figure-4:**
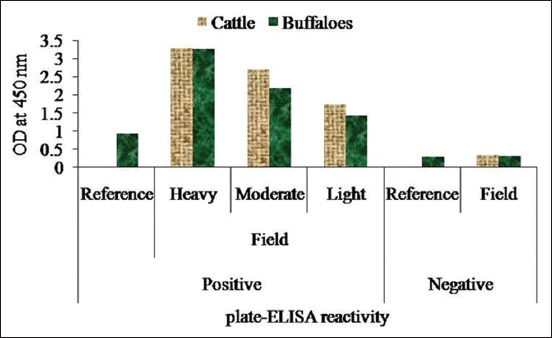
Plate enzyme-linked immunosorbent assay reactivity pattern of *Fasciola* *gigantica* infection.

The integrity of the procured native CP of *F*. *gigantica* revealed a single observable band on the 10% SDS-PAGE, which was used to determine the anti-*F*. *gigantica* antibody in the cattle and buffaloes. There was no cross-reaction of the CP antigen with *G*. *crumenifer* field sera at any point of time in plate-/dot-ELISA. Out of 150 screened bovine, sera of 47 animals were found to be reactive in dot-ELISA with 31.25 and 31.58% prevalence rate in cattle and buffaloes, respectively (Figure-1). The *F*. *gigantica* seropositive bovines with heavy, moderate, and light level of infection were 44.68%, 34.04%, and 21.28%, respectively (p<0.05 between heavy and light; p>0.05 between moderate and heavy/light) (Figure-2). The % distribution of *F*. *gigantica* seropositive/negative bovines was depicted in Figure-3. The OD at 450 nm of pooled sera of seropositive cattle with heavy, moderate, and light reactivity in plate-ELISA was 3.2845, 2.7135, and 1.7225, respectively, which was significantly higher with field negative sera (Figure-4). Likewise, OD at 450 nm of pooled sera of seropositive buffaloes with heavy, moderate, and light reactivity in plate-ELISA was 3.26375, 2.18375, and 1.433, respectively, which was significantly higher with reference/field negative sera (Figure-4).

## Discussion

The field applicability of the diagnostic assay is the final goal and worthwhile. Several immunodiagnostic assays were tried for the prepatent detection of fasciolosis in field setup, namely, double immunodiffusion [21], skin hypersensitivity reactions [22], dot-ELISA [23], and dot immunoperoxidase [24] with their different acceptability levels. The dot-ELISA is more widely accepted, since the test is easy to conduct, and results can be ascertained rapidly.

Realizing the immense potentiality of the test and the laboratories all around the world have standardized a CP-based dot-ELISA for the early diagnosis of fasciolosis in human and ruminants with high level of sensitivity/specificity (~100%) in experimental conditions while a little bit compromised sensitivity/specificity in field conditions and recommended to use it in the field situations [16,17,25]. The 28 kDa CP observed high-level of sensitivity in the ELISA for the detection of experimental *F*. *gigantica* infection in buffaloes by 3-4 WPI [12,16,17,20]. The integrity of the selected CP antigen on SDS-PAGE confirmed that the antigen was made up of a single visible band, as reported by earlier scientists. In view of the absence of the reference test for the diagnosis of tropical fasciolosis, PM data on parasite prevalence would have been served in better way for the calculation of the sensitivity/specificity, but by knowing the fact of the vegetarian nature of the human population of the study area, there is no any recognized slaughterhouse of the large ruminants, so we were unable to calculate the sensitivity/specificity of the test and antigen in the field conditions. On the other hand, the trematodes are well known for its intermittent egg shedding behavior, and further, there is no any direct correlation between the egg per gram of feces and actual parasitic load, so the use of coprological data for evaluating serological test is worthless [26]. Interestingly, Raina *et al*. [20] correctly pointed out that the sensitivity of the serological assay was determined by the antibody response elicited by the individual host rather the fluke burden. Hence, it is the demand of the time to describe a reference/gold standard test for the calculation of sensitivity/specificity of the new upcoming serological/molecular test for the detection of *F*. *gigantica* infection.

Overlapping antigens in the trematodes frequently observed cross-reactivity among themselves [13]; fortunately, in the present study, no cross-reaction was noted with the rumen amphistome, *G*. *crumenifer*, and few more researcher observed no cross-reaction with *Paramphistomum epiclitum*, *Gigantocotyle explanatum*, hydatid cyst, and *Strongyloides papilossus* [14,15,20].

The present study recorded moderate level of prevalence (31.33%) (Figures-1-3) of *F*. *gigantica* seropositive animal, which seems to be due to heavy rainfall resulting into plenty of water body harboring the snail intermediate host. Depending on the agroclimatic conditions and the type of the ruminant host, the researcher all over the world time to time reported the low-to-high level of prevalence of *F*. *gigantica* using specific diagnostic antigen in the ELISA [10,12,18,27,28]. The water-loving nature of the buffaloes could be hypothesized that they can have more incidence rate of *F*. *gigantica* over the cattle. Interestingly, in the current work, there was non-significant (p>0.05) difference in the seroprevalence rate of tropical liver fluke between cattle and buffaloes (Figure-1), and this might be due to the adoption of the same pattern of rearing of the large ruminants by the farmers in the study area [29].

The reactivity observed in plate-ELISA in terms of OD values at 450 nm between the positive and reference/field negative sera in both cattle and buffaloes was significantly (p<0.05) different (Figure-4). The moderately reacted bovine in dot-/plate-ELISA fall at the borderline and can be shifted toward the up-/down-side of the reactivity of *F*. *gigantica* infection [14,15].

In spite of many merits of CP-based ELISA, the one major inherited demerit is that being the antibody detection test, unable to differentiate the active and passive infection status of the animals. Hence, we have to consider the clinical status of the animals to ascertain the final infection status of the animals [30,31].

## Conclusion

Overall, we pass our recommendation to the said laboratory to move forward and come up with a first field-based detection kit for the tropical fasciolosis using the CP antigen in paper-/dot-ELISA, which will be very useful for field veterinarians for quick and timely detection of the animals requiring urgent therapy with the flukicide at the farmer’s doorstep.

## Authors’ Contributions

NK planned and accomplished the overall research work. AV collected the sera and did the ELISA. NK did the data analysis and drafted the manuscript. JBS revised the manuscript. All authors read and approved the final manuscript.

## References

[1] Gupta S.C, Ghosh S, Raina O.K, Joseph D, Preeti R, Singh B.P, Mishra A.K, Chandra D, Samanta S (2008). Status and prevalence of fasciolosis in cattle and buffaloes in different agro-climatic zones of Uttar Pradesh. J. Vet. Parasitol.

[2] Pandya S.S, Hasnani J.J, Patel P.V, Chauhan V.D, Hirani N.D, Shukla R, Dhamsaniya H.B (2015). Study on prevalence of fasciolosis in buffaloes at Anand and Ahmedabad districts, Gujarat, India. Vet. World.

[3] Elelu N, Ambali A, Coles G.C, Eisler M.C (2016). Cross-sectional study of *Fasciola gigantica* and other trematode infections of cattle in Edu local government area, Kwara state, North-Central Nigeria. Parasit. Vectors.

[4] Mulcahy G, Dalton J.P (2001). Cathepsin-L proteinases as vaccines against infection with *Fasciola hepatica*(liver fluke) in ruminants. Res. Vet. Sci.

[5] WAAVP Congress (2005). *Fasciola hepatica*:Suppression of Host Immune Responses and Susceptibility of Other Diseases. Annual Convention of the World Association for the Advancement of Veterinary Parasitology.

[6] Sariözkan S, Yalçin C (2011). Estimating the total cost of bovine fasciolosis in Turkey. Ann. Trop. Med. Parasitol.

[7] Jean-Richard V, Crump L, Abicho A.A, Naré N.B, Greter H, Hattendorf J, Schelling E, Zinsstag J (2014). Prevalence of *Fasciola gigantica* infection in slaughtered animals in south-eastern Lake Chad area in relation to husbandry practices and seasonal water levels. BMC Vet. Res.

[8] Fairweather I (2005). Triclabendazole:New skills to unravel an old(ish) enigma. J. Helminthol.

[9] Gupta S.C, Yadav S.C (1992). Sexual maturity of *Fasciola gigantica* in experimentally infected rabbits, goats and buffaloes. Indian J. Parasitol.

[10] Rehman T, Khan M.N, Abbas R.Z, Babar W, Sikandar A, Zaman M.A (2016). Serological and coprological analyses for the diagnosis of *Fasciola gigantica* infections in bovine hosts from Sargodha, Pakistan. J. Helminthol.

[11] Valero M.A, Bargues M.D, Khoubbane M, Artigas P, Quesada C, Berinde L, Ubeira F.M, Mezo M, Hernandez J.L, Agramunt V.H, Mas-Coma S (2016). Higher physiopathogenicity by *Fasciola gigantica* than by the genetically close *F. hepatica*:Experimental long-term follow-up of biochemical markers. Trans R. Soc. Trop. Med. Hyg.

[12] Varghese A, Raina O.K, Nagar G, Garg R, Banerjee P.S, Maharana B.R, Kollannur J.D (2012). Development of cathepsin-L cysteine proteinase based dot-enzyme-linked immunosorbent assay for the diagnosis of *Fasciola gigantica* infection in buffaloes. Vet. Parasitol.

[13] Yokananth S, Ghosh S, Gupta S.C, Suresh M.G, Saravanan D (2005). Characterization of specific and cross-reacting antigens of *Fasciola gigantica* by immunoblotting. Parasitol. Res.

[14] Kumar N, Ghosh S, Gupta S.C (2008). Detection of *Fasciola gigantica* infection in buffaloes by enzyme-linked immunosorbent assay. Parasitol. Res.

[15] Kumar N, Ghosh S, Gupta S.C (2008). Early detection of *Fasciola gigantica* infection in buffaloes by enzyme-linked immunosorbent assay and dot enzyme-linked immunosorbent assay. Parasitol. Res.

[16] Yadav S.C, Saina M, Raina O.K, Nambi P.A, Jadav K, Sriveny D (2005). *Fasciola gigantica* cathepsin-L cysteine proteinase in the detection of early experimental fasciolosis in ruminants. Parasitol. Res.

[17] Sriveny D, Raina O.K, Yadav S.C, Chandra D, Jayraw A.K, Singh M, Velusamy R, Singh B.P (2006). Cathepsin-L cysteine proteinase in the diagnosis of bovine *Fasciola gigantica* infection. Vet. Parasitol.

[18] Avcioglu H, Guven E, Balkaya I, Kaynar O, Hayirli A (2014). Evaluation of coprological and serological techniques for diagnosis of bovine fasciolosis. Isr. J. Vet. Med.

[19] Yadav P, Singh R (2011). A review on anthelmintic drugs and their future scope. Int. J. Pharm. Pharm. Sci.

[20] Raina O.K, Yadav S.C, Sriveny D, Gupta S.C (2006). Immuno-diagnosis of bubaline fasciolosis with *Fasciola gigantica* cathepsin-L and recombinant cathepsin L 1-D protease. Acta Trop.

[21] Ouchterlony O, Ackroyd J.F (1964). Gel diffusion technique. Immunological Methods.

[22] Doyle J.J (1973). Skin hypersensitivity reaction induced in calves by experimental infections with *Fasciola hepatica*. Int. Arch. Allergy.

[23] Pappas M.G, Hajkowski R, Hockmeyer W.T (1983). Dot-enzyme linked immunosorbent assay (dot-ELISA):A micro technique for the rapid diagnosis of visceral leishmaniasis. J. Immunol. Methods.

[24] Maisonnave J (1999). Standardization of dot immuno-peroxidase assay for field diagnosis of *Fasciola hepatica* infected cattle. Vet. Parasitol.

[25] Cornelissen J.B.W, Gassenbeek C.P.H, Borgsteede F.H.M, Holland W.G, Harmsen M.M, Bocrsma W.J.A (2001). Early immune-diagnosis of fasciolosis in ruminants using recombinant *Fasciola hepatica* cathepsin-L-like protease. Int. J. Parasitol.

[26] Fagbemi B.O, Guobadia E.E (1995). Immuno-diagnosis of fasciolosis in ruminants using a 28 kDa cysteine protease of *Fasciola gigantica* adult worms. Vet. Parasitol.

[27] Damwesh S.D, Ardo M.B (2013). Detection of *Fasciola gigantica* antibodies using pourquier ELISA kit. Sokoto J. Vet. Sci.

[28] Chamuah J.K, Jacob S.S, Sakhrie A, Borkotoky D (2014). Serological prevalence of *Fasciola gigantica* in mithun (*Bos frontalis*). Int. J. Livest. Res.

[29] Sabapara G.P, Desai P.M, Kharadi V.B, Saiyed L.H, Singh R.R (2010). Housing and feeding management practices of dairy animals in the tribal area of South Gujarat. Indian J. Anim. Sci.

[30] Palmer D.G, Lyon J, Palmer M.A, Forshaw D (2014). Evaluation of a copro-antigen ELISA to detect *Fasciola hepatica* infection in sheep, cattle and horses. Aust. Vet. J.

[31] Abdolahi-Khabisi S, Sarkari B (2016). Detection of *Fasciola hepatica* and *Fasciola gigantica* common and uncommon antigens, using rabbit hyper immune serum raised against their excretory-secretory and somatic antigens. J. Parasit. Dis.

